# China-origin G1 group isolate FPV072 exhibits higher infectivity and pathogenicity than G2 group isolate FPV027

**DOI:** 10.3389/fvets.2024.1328244

**Published:** 2024-01-15

**Authors:** Qiaoqiao Xie, Zhen Sun, Xiu Xue, Yajie Pan, Shuye Zhen, Yang Liu, Jiuyu Zhan, Linlin Jiang, Jianlong Zhang, Hongwei Zhu, Xin Yu, Xingxiao Zhang

**Affiliations:** ^1^School of Life Sciences, Ludong University, Yantai, China; ^2^Collaborative Innovation Center for the Pet Infectious Diseases and Public Health in the Middle and Lower Stream Regions of the Yellow River, Yantai, China; ^3^Provincial Engineering Research Center for Pet Animal Vaccines, Yantai, China

**Keywords:** feline parvovirus (FPV), VP2, infectivity, pathogenicity, China

## Abstract

**Introduction:**

Feline parvovirus (FPV), a single-stranded DNA virus, is accountable for causing feline panleukopenia, a highly contagious and often lethal disease that primarily affects cats. The epidemiology prevalence and pathogenicity of FPV in certain regions of China, however, remains unclear. The aim of this research was to investigate the epidemiology of FPV in different regions of China in 2021 and compare its infectivity and pathogenicity.

**Methods:**

In this research, a total of 36 FPV strains were obtained from diverse regions across China. Phylogenetic analysis was performed based on the VP2 and NS1 sequences, and two representative strains, FPV027 and FPV072, which belonged to different branches, were selected for comparative assessment of infectivity and pathogenicity.

**Results and discussion:**

The results revealed that all strains were phylogenetically classified into two groups, G1 and G2, with a higher prevalence of G1 strains in China. Both *in vitro* and *in vivo* experiments demonstrated that FPV072 (G1 group) exhibited enhanced infectivity and pathogenicity compared to FPV027 (G2 Group). The structural alignment of the VP2 protein between the two viruses revealed mutations in residues 91, 232, and 300 that may contribute to differences in infectivity and pathogenicity. The findings from these observations will contribute significantly to the overall understanding of the molecular epidemiology of FPV in China and facilitate the development of an effective FPV vaccine.

## 1 Introduction

Feline parvovirus (FPV), alternatively termed feline panleukopenia virus (FPLV), is the etiological agent for feline panleukopenia (FPL) in cats, exhibiting a predilection for kittens. This virus demonstrates cross-species infectivity, capable of infecting various members of the felid family, encompassing both wild and domestic species (1–3). Moreover, it has the potential to affect certain species belonging to the Mustelidae, Procyonidae, and Viverridae families, such as raccoons, ring-tailed cats, foxes, and minks (4). FPL is a highly infectious and frequently lethal disease that is distinguished by pancytopenia, acute and severe inflammation of the intestines, dehydration, and sepsis caused by lymphoid depletion (5). FPV has a genome length of about 5.1 kilobases and contains two open reading frames (ORFs), namely ORF1 and ORF2. The ORF1 gene contains two non-structural proteins, specifically NS1 and NS2. The NS1 protein includes a domain that encodes a polyprotein, a domain that binds to the origin of replication (ORI), a helicase domain, and functional domains for transactivation. These domains are crucial for controlling replication, DNA packaging, cytotoxicity, and pathogenicity. On the other hand, the NS2 protein is encoded by combining a 260-nt fragment from the left side with a 238-nt fragment from the right side, both originating from the NS1 open reading frame (6–8). Two capsid proteins, VP1 and VP2, are encoded by the ORF2 region. Encoded by alternative splicing, the VP1 protein includes the entire VP2 protein sequence and possesses a distinct N-terminal sequence of 143 residues, which impacts the transportation of capsids to the nucleus and the infection of cells (9). The VP2 protein constitutes approximately 90% of the viral capsid, making it the predominant capsid protein. It serves as the primary target for neutralizing antibodies against parvovirus and plays a crucial role in determining hemagglutination, cellular tropism, and host range (10). Specific amino acids within the VP2 protein are essential for antigenicity, pathogenicity, and host range determination, and subsequently influencing the viral surface structure (11). The amino acids 80K, 564N, and 568A are critical for FPV replication in cats, and the key amino acids residues (80K, 93K, 103V, 323D, 564N, and 568A) has been widely used to distinguish canine parvovirus type 2 (CPV-2) from FPV (12).

The majority of FPV vaccines are modified live virus vaccines (MLV) that have been passaged numerous times on feline or mink cell lines. Inactivated vaccines were used extensively in the past but provide limited amounts of antibodies that protect for a short time, and have been largely replaced by MLVs (13). Although, commercial FPV vaccines have been extensively employed on a global scale, exhibiting promising outcomes in curtailing the transmission of the viral ailment and yielding substantial reductions in both morbidity and mortality rates, FPV is still prevalent in various countries globally (14–18). There are few studies on the molecular epidemiology of FPV in China, and the epidemic situation and molecular characteristics of epidemic strains are still unclear (6, 12, 19). The objectives of the study were to clarify molecular characterization of the epidemic FPV isolated from the domestic cats in parts of China, and to investigate the pathogenicity of the isolates in cats.

## 2 Materials and methods

### 2.1 Sample collection

A total of 167 clinical samples (feces or rectal swabs) were collected from pet cats exhibiting one or more clinical signs of parvovirus infection (lethargy, anorexia, vomiting, retching, and/or diarrhea). The cats were subsequently confirmed to be infected with FPV in veterinary hospitals which were located in different regions in China: Linyi (*n* = 25), Weifang (*n* = 15), Rizhao (*n* = 21), Shanghai (*n* = 35), Changsha (*n* = 20) and unidentified areas (*n* = 51). The presence of FPV in these samples had been confirmed to be positive for FPV in veterinary hospital and rechecked using antigen rapid test kit (Colloidal Gold-Based) (Quicking Biotech, Shanghai, China) in the laboratory. After being mixed in 1 mL of 0.1 M PBS (pH 7.4), the samples were then subjected to centrifugation at 10,000 g for 10 min at a temperature of 4°C, and stored at −80°C until use. Four to six FPV isolates from each area and 10 isolates from unidentified areas (36 strains in total) were chosen for subsequent analysis. The details are summarized in Table 1.

**Table 1:** Detailed description of feline panleukopenia virus (FPV) characterized in this study.

Strains	VP2 Genbank No.	NS1 Genbank No.	Location	Date	Age (month)	Gender^a^	Vaccination status	Number
FPV003	OQ398386	OQ398423	Linyi, Shandong	10/24/2021	11	Ma	Vaccinated	5
FPV008	OQ398387	OQ398424		10/27/2021	4	Ma	Unvaccinated	
FPV013	OQ398388	OQ398425		10/28/2021	4	Ma	Unvaccinated	
FPV014	OQ398389	OQ398426		10/28/2021	8	Fe	Unvaccinated	
FPV021	OQ398390	OQ398427		10/28/2021	24	Fe	Unvaccinated	
FPV046	OQ398418	OQ398455	Weifang, Shandong	10/25/2021	10	Fe	Vaccinated	4
FPV048	OQ398419	OQ398456		11/1/2021	2	Fe	Vaccinated	
FPV049	OQ398420	OQ398457		11/2/2021	2	Fe	Unvaccinated	
FPV050	OQ398421	OQ398458		11/3/2021	12	Ma	Unvaccinated	
FPV036	OQ398396	OQ398433	Rizhao, Shandong	11/7/2021	48	Fe	Vaccinated	6
FPV037	OQ398397	OQ398434		11/6/2021	3	Fe	Unvaccinated	
FPV038	OQ398398	OQ398435		11/7/2021	36	Fe	Unvaccinated	
FPV039	OQ398399	OQ398436		11/8/2021	36	Fe	Unvaccinated	
FPV040	OQ398400	OQ398437		11/8/2021	60	Fe	Vaccinated	
FPV055	OQ398422	OQ398459		11/8/2021	12	Ma	Vaccinated	
FPV026	OQ398391	OQ398428	Shanghai	11/5/2021	5	Fe	Unvaccinated	5
FPV027	OQ398392	OQ398429		11/5/2021	2	Ma	Unvaccinated	
FPV028	OQ398393	OQ398430		11/5/2021	8	Ma	Unvaccinated	
FPV029	OQ398394	OQ398431		11/5/2021	36	Ma	Vaccinated	
FPV030	OQ398395	OQ398432		11/5/2021	10	Fe	Vaccinated	
FPV041	OQ398401	OQ398438	Changsha, Hunan	12/31/2021	60	Ma	Unvaccinated	6
FPV042	OQ398402	OQ398439		12/30/2021	48	Fe	Unvaccinated	
FPV043	OQ398403	OQ398440		12/30/2021	48	Ma	Unvaccinated	
FPV044	OQ398404	OQ398441		12/29/2021	12	Ma	Vaccinated	
FPV045	OQ398405	OQ398442		12/31/2021	3	Fe	Unvaccinated	
FPV072	OQ398406	OQ398443		12/31/2021	9	Fe	Vaccinated	
FPV088	OQ398415	OQ398452	Unidentified regions	11/11/2021	5	Fe	Vaccinated	10
FPV086	OQ398413	OQ398450		11/11/2021	7	Ma	Unvaccinated	
FPV090	OQ398417	OQ398454		12/11/2021	3	Ma	Unvaccinated	
FPV089	OQ398416	OQ398453		11/11/2021	7	Ma	Unvaccinated	
FPV085	OQ398412	OQ398449		11/12/2021	12	Fe	Unvaccinated	
FPV087	OQ398414	OQ398451		11/12/2021	15	Fe	Vaccinated	
FPV077	OQ398408	OQ398445		11/12/2021	24	Fe	Vaccinated	
FPV080	OQ398409	OQ398446		11/11/2021	10	Ma	Unvaccinated	
FPV076	OQ398407	OQ398444		11/10/2021	10	Fe	Vaccinated	
FPV081	OQ398410	OQ398447		11/11/2021	24	Ma	Unvaccinated	
Total								36

a Ma means male, and Fe means female.

### 2.2 PCR and DNA sequencing assays

For each sample, the viral DNA was extracted by the EasyPure Viral DNA/RNA Kit (TransGen Biotech, Beijing, China) according to the manufacturer’s instructions. Presence of FPV in extracted viral DNA was further confirmed by PCR, targeting the 681 bp VP2 fragments of FPV (Supplementary Table S1). In accordance with the guidelines provided by the manufacturer, PCR was performed utilizing PrimeSTAR^®^ Max DNA Polymerase (Takara Biotech, Dalian, China) in a total volume of 20 μL, which consisted of 10 μL of PrimeSTAR Max Premix (2×), 0.8 μL of each primer (10 μM), 1 μL of DNA, and 7.4 μL of sterile distilled water. The PCR condition was set as an initial denaturation step at 98°C for 1 min; 35 cycles at 98°C for 10 s, annealing at 57°C for 5 s, extension at 72°C for 50 s; and a final extension at 72°C for 10 min. Afterwards, the positive samples underwent amplification of the entire NS1 and VP2 genes by utilizing two sets of designed primers, NS1-F/R or VP2-F/R (Supplementary Table S1), in a final volume of 50 μL comprising 25 μL of PrimeSTAR Max Premix (2×), 2 μL of each primer (10 μM), 5 μL of DNA, and 16 μL of sterile distilled water. The PCR condition was set as an initial denaturation step at 98°C for 1 min; 35 cycles at 98°C for 10 s, annealing at 57°C for 15 s, extension at 72°C for 2 min 30 s; and a final extension at 72°C for 10 min. Positive amplicons were sent for direct Sanger sequencing to BGI Biotech in Beijing.

### 2.3 Sequence and phylogenetic analyses

BioEdit version 7.2.5 was used to build sequences in accordance with an overlapping technique. The sequences generated were aligned and compared with FPV sequences from the NCBI database using version 7.0 of the MEGA software package. With MEGA7.0, the phylogenetic relationships were assessed. Maximum-likelihood (ML) methods were utilized to construct the phylogenetic trees. The bootstrap values were produced using a total of 1,000 replicates. Thirty-six NS1 gene sequences and 38 VP2 gene sequences were acquired from GenBank as reference sequences in order to conduct a more thorough analysis.

### 2.4 Virus isolation

Feline kidney cells CRFK (Procell, Wuhan, China) were cultured with DMEM (Vivacell, Shanghai, China) containing 10% fetal bovine serum (FBS) (ZETA, San Francisco, United States) and 1% penicillin-streptomycin. When cultures reached 80%–90% confluence, subculture was performed. Before being infected with virus, CRFK were digested and suspended in serum-free DMEM at a concentration of 5 × 10^5^/mL. The virus sample supernatants were sterilized by filtration with a 0.22 μm microporous filter, synchronously inoculated into cells at a volume ratio of 1:100, gently mixed and incubated at 37°C with 5% CO_2_ concentration. After 1 h, the virus supernatant was discarded and substituted with the complete cell culture medium. Following a 72 h culture period, the cells were injected with the freeze-thawed culture media and refrozen twice more in a refrigerator at −80°C. After 3–4 generations of continuous blind passage, the observation of cytopathic effect (CPE) was noted. The CPE which is typical of FPV infection often involves total destruction of the cell monolayer. Earlier CPE evidence includes cell rounding and disassociation of the cells from the flask surface (20).

### 2.5 Virus titration and growth kinetics

Viral titration was assessed using the 50% cell culture infectious dose (TCID_50_) assay. Briefly, 100 μL CRFK cell suspension, containing 3 × 10^4^ cells in DMEM with 10% FBS and 1% penicillin-streptomycin, was seeded into each well of a 96-well plate and kept at 37°C for incubation. After 24 h incubation, CRFK cells were inoculated with 10-fold dilutions of the virus for 1 h, followed by washing and then incubation with DMEM with 2% FBS and 1% penicillin-streptomycin for 3–4 days and checked daily for CPE. TCID_50_ end-point titers were calculated using the Reed and Muench method. Virus growth kinetics were determined by testing viral titers after three consecutive freeze-thaw cycles.

### 2.6 Western blot assay

The FPV-infected and mock cells were lysed in Laemmli sample buffer, boiled for 10 min. Proteins were transferred utilizing the Trans-Blot^®^ Turbo^TM^ Transfer System (Bio-Rad Laboratories, Hercules, CA, United States) from gels onto PVDF membranes. Membranes were blocked with PBS, 5% (w/v) skim milk, and 0.5% (v/v) Tween 20 for 2 h at room temperature and incubated with mouse monoclonal antibody recognizing CPV VP2 protein (Abcam, Cambridge, United Kingdom) (9, 21) at 4°C overnight. Following multiple rinses with PBS-0.05% Tween 20, the membranes were then incubated at 37°C for 1 h with HRP-conjugated goat anti-mouse IgG (Abcam, Cambridge, United Kingdom). Afterwards, the membranes were thoroughly rinsed with PBS-0.05% Tween-20 and treated with Pierce ECL Plus Western Blotting (Thermo Fisher Scientific, MA, United States) for visualization.

### 2.7 Transmission electron microscope

The harvested cell cultures were partially purified by ultracentrifugation through a 15% (w/v) sucrose cushion at a speed of 45,000 g for 3 h at 4°C. After purification, the sample was treated with a solution of alcohol saturated with 2% uranyl acetate and a solution of 2.6% lead citrate, and observed using a transmission electron microscope (H-7800, Hitachi, Japan).

### 2.8 Immunofluorescence assay

CRFK cells infected with FPV isolate (MOI = 0.1) in 96-well plates were treated with cold 80% (v/v) acetone for 15 min, washed with PBS three times, exposed to a mouse anti-CPV antibody (Abcam, Cambridge, United Kingdom) at 37°C for 1 h, and then treated with an Alexa Fluor 488-conjugated goat anti-mouse IgG H&L antibody (Abcam, Cambridge, United Kingdom). Then, diamidino-2-phenylindole (DAPI) (Solarbio, Beijing, China) dye was added for fluorescent staining of cell nuclei. FPV infected cells were observed under a fluorescence microscope (ZEISS, Oberkochen, Germany) after being washed with PBS; cells evidencing intranuclear fluorescence were considered infected with FPV.

### 2.9 Animal experiments

Fifteen conventional British Shorthair pet kittens used in the challenge experiments were purchased from Jianyouda Biomedical Technology Co., Ltd. The kittens were brought to the facility when they were between 8 and 10 weeks old. They were acclimated and used in the studies at the age of 12–14 weeks. The animals were kept in separate groups in a closed facility. PCR and/or ELISA tests confirmed that the cats were free from feline herpesvirus (FHV), feline calicivirus (FCV), feline immunodeficiency virus (FIV), and feline leukaemia virus (FELV). Kittens were intraperitoneally challenged with 10^4.7^ TCID_50_ of the viruses FPV027 (Group 1, *n* = 5) and FPV072 (Group 2, *n* = 5), respectively. The kittens in the mock control group were given DMEM intraperitoneally (Group 3, *n* = 5). The duration of the clinical observation period amounted to 14 days. Throughout this period, daily clinical examinations were conducted on the kittens (22). Briefly, scoring was based on attitude (0–3 points), appetite (0–3 points), vomiting (0–3 points), and fecal appearance (0–4 points). The clinical score was given by the sum of the 4 assigned values. The severity of the values is reported in ascending order. In addition, the white blood cell (WBC) count was recorded every 2 days. Kittens showing signs of terminal FPV were euthanized in order to avoid unnecessary suffering. All animal experiments were approved and supervised by the Institutional Animal Care and Use Committee (IACUC) of Ludong University.

### 2.10 Detection of virus shedding

In order to ascertain the shedding of the virus, samples of feces were collected and immersed in 500 mL of phosphate buffered saline (PBS). After vortexing and 30 min incubation, the swabs were removed, and the extract was centrifuged at 1,000 g for 10 min to get rid of debris. PCR was performed using the extracted viral DNA from the supernatant. The genome copy numbers of FPV were quantified using the Feline Parvovirus Real Time PCR detection kit (TIANDZ, Beijing, China).

### 2.11 Pathology, histopathology and immunohistochemical staining

Pathological examination was conducted on all animals that perished or were euthanized during the experiment. Carcasses were dissected within a span of 2 h after the demise. Samples extracted from the intestinal tract and spleen were embedded in paraffin wax after being fixed in 8% formaldehyde. The blocks were sectioned at 4–6 mm and the sections were stained with haematoxylin-eosin and examined under light microscope. Intestinal immunohistochemistry was then scored according to the description in the previous literature (23). In brief, the ratio of apical denudation of villi between 0% and 10%, scored 3; between 11% and 40%, scored 2; between 41% and 70%, scored 1; between 71% and 100%, scored 0. The histopathologically examined organs were subsequently subjected to immunohistochemical analysis. A primary antibody specific to parvovirus (Abcam, Cambridge, United Kingdom) was utilized, followed by a secondary antibody conjugated with HRP and specific for mouse IgG (H + L) (Thermo Fisher Scientific, MA, United States). The binding events were visualized using the peroxidase anti-peroxidase method with diaminobenzidine as the chromogen.

### 2.12 Molecular dynamics simulation

The initial structure of the VP2 of FPV072 and FPV027 were predicted with trRosetta online server (24).^1^ Using H++ server, the protonation states of ionizable residues were determined at pH = 7.0 (25). In FPV072 VP2, His102 and His are protonated at Nδ atom; His70, His234, and His483 are deprotonated; the carboxyl groups in Asp373, Asp375, and Glu368 are all protonated. In FPV027 VP2, the protonation state of titrable amino acid residues is almost the same as FPV072 VP2, except for the Nδ protonation of His384. Hydrogen atoms and missing atoms were subsequently added to the complex structures using the t-Leap procedure of AMBER22. To keep the whole system electric neutral state, five Na^+^ ions were introduced to the systems built above. Then, each system was solvated in an octahedron box with a 10.0 Å distance around the solute.

The ff14SB force field was adopted for the proteins (26). AMBER22 software package was utilized to conduct the molecular dynamics simulations (MD simulations). To minimize water molecules, a total of 5,000 cycles of steepest descent followed by 5,000 cycles of conjugated gradient minimizations were conducted. And then, the minimizations were repeated to minimize the whole system. Thirdly, each system was gradually heated from 0 K to its physiological temperature over a period of 300 ps. After that, 500 ps equilibrium simulation was performed to balance the density. Finally, 200 ns MD simulation was performed for each system under NPT condition to produce trajectory. The particle mesh Ewald (PME) method (27) was used to evaluate long-range electrostatic interactions, while the short range interactions were cut off at 10.0 Å. The integration step was set to 2 fs in all the simulations.

### 2.13 Statistical analysis

The analysis of the data was conducted using GraphPad Prism Software 9.5 (GraphPad Software, SanDiego, CA, United States). Results were represented as mean ± SEM, and one-way analysis of variance (ANOVA) was used to compare data among three or more groups; *p* < 0.05 was considered as statistically significant, ^*^p < 0.05, ^**^p < 0.01.

## 3 Results

### 3.1 Isolation and identification of FPV strains

**Figure 1:**
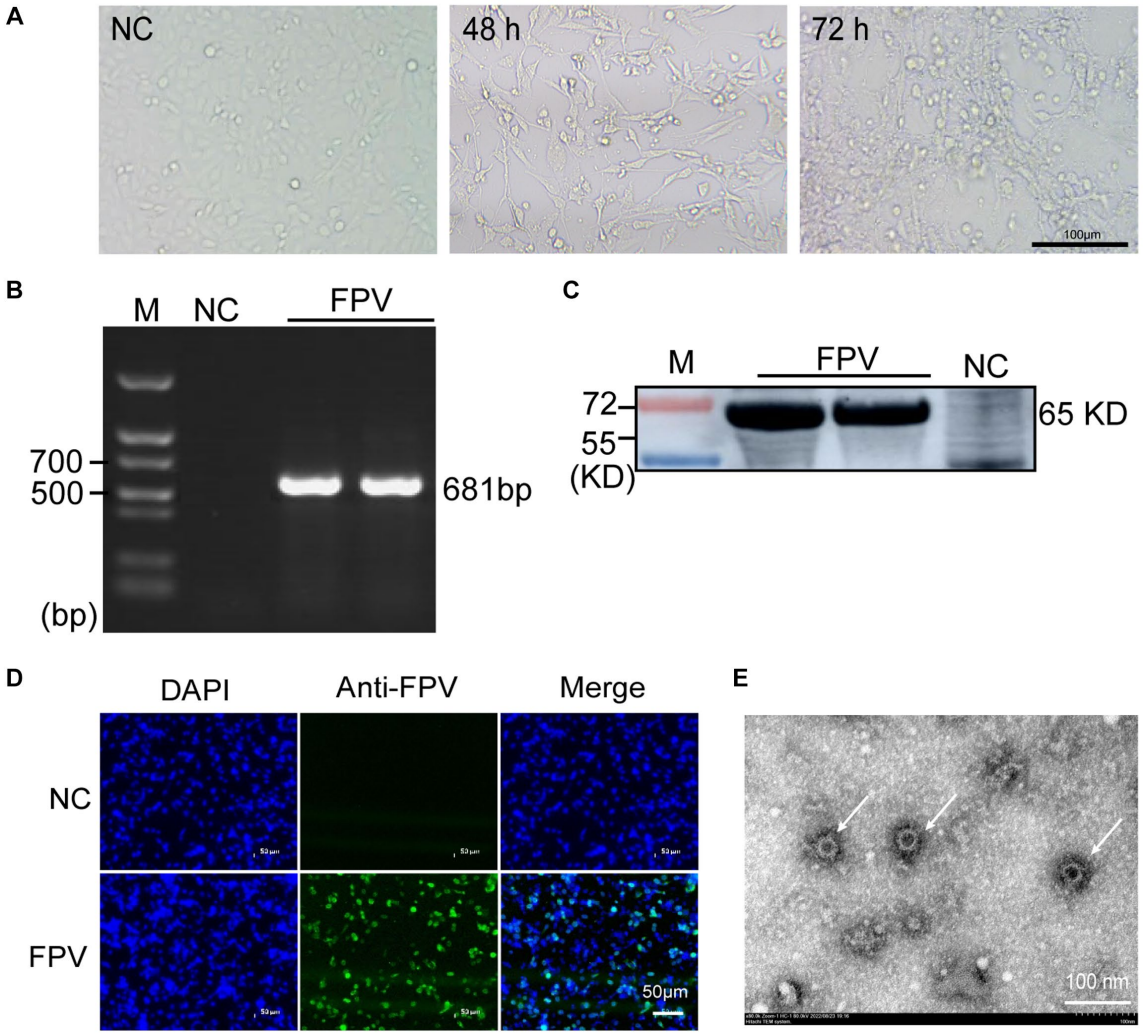
Isolation and identification of FPV in CRFK cells. **(A)** Cytopathic effect (CPE) of CRFK cells infected with FPV at 48 hpi and 72 hpi (×100). The negative control cells were mock infected with FPV (×100). Scale bar = 100 μm. **(B)** PCR analysis of the total cell DNA from the third passages of virus-infected cells or mock-infected cells. **(C)** Western blot analysis of the total cell lysates from virus-infected cells using anti-CPV polyclonal antibodies. **(D)** Immunofluorescence detection results at 24 h after inoculation. Scale bar = 50 μm. **(E)** Electron micrograph of FPV virions (arrow heads) in cell culture media of infected CRFK cells. Scale bar = 100 nm.

CRFK cells were inoculated with FPV-positive samples at a 100-fold dilution, and visible CPE was observed 48 h after inoculation (Figure 1A). Viral supernatants and cell lysates were collected and subjected to both PCR and immunoblotting to test for virus production. As expected, PCR amplification of the viral genomic DNA revealed a specific band of 681 bp corresponding to the partial amplification of the VP2 gene (Figure 1B). Representative WB bands for FPV were detected in infected cells (Figure 1C). Furthermore, FPV antigen was detected by IFA using a parvovirus-specific mouse monoclonal antibody and cell nuclei was detected by DAPI. Cells expressing FPV antigens were identified by the specific green cytoplasmic signals. No positive staining was observed in uninfected cells (Figure 1D). FPV particles in culture media and infected cells were imaged with a transmission electron microscope (TEM). Typical non-enveloped round virus particles were observed with diameters of 20–25 nm (Figure 1E). The 36 strains of FPV (Table 1) were isolated and identified according to the above methods.

### 3.2 Genetic evolution and amino acid mutations of VP2

**Figure 2:**
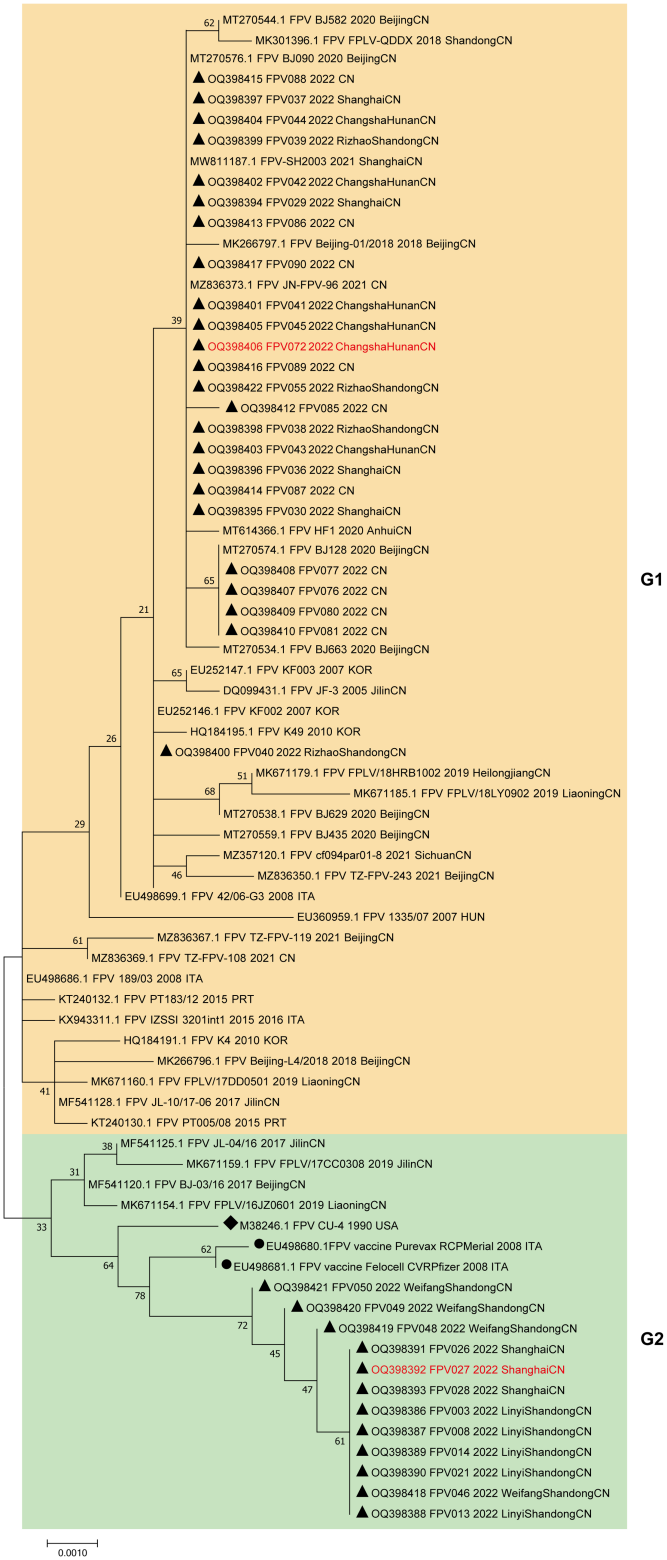
Phylogenetic tree analysis of the VP2 nucleotide sequences of the FPV strains. A phylogenetic tree based on 36 VP2 gene sequences from domestic cats and 38 reference FPV strains was constructed by the maximum likelihood method using the MEGA software, version 7.0. ▲ Indicates the FPV strains in this study. ● Indicates the FPV vaccine strains. ◆ Indicates the FPV standard strain. Horizontal branch lengths are proportional to genetic distances. Scale bars indicate nucleotide substitutions per site. Bootstrap values were calculated based on 1,000 replicates.

To further examine the relationship between the 36 strains with FPV strains in other regions of the world, we reconstructed a phylogenetic tree containing 38 FPV reference sequences and the 36 cat-derived parvovirus sequences based on the complete VP2 gene sequence (Figure 2). The phylogenetic tree was divided into two groups, G1 and G2. Strains in G1 mostly comprised FPV isolates from Asia (China and Korea) and a few from Europe (Italy and Portugal). Strains in G2 consisted almost exclusively of Chinese isolates, and included two vaccine strains, indicating a close relationship between G2 strains and vaccine strains. A total of 24 of the 36 FPV strains (66.7%) were distributed in G1, suggesting that G1 strains were the predominant strains circulating in China.

**Figure 3:**
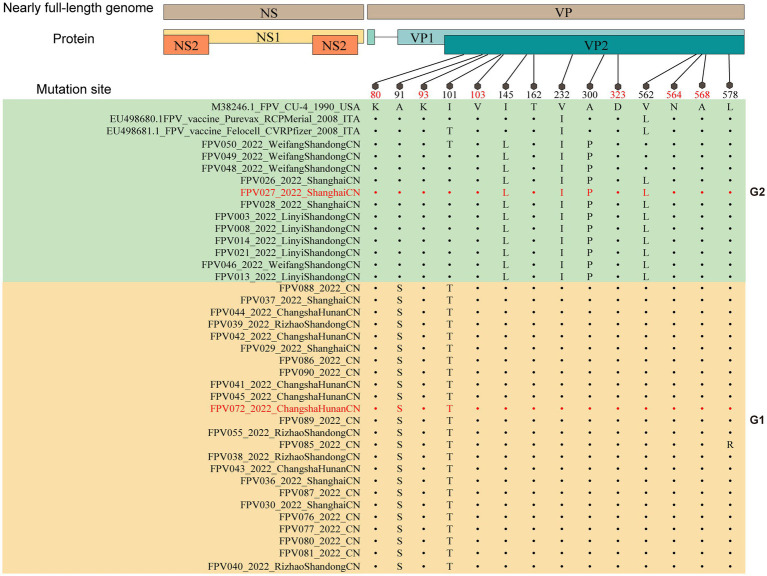
Molecular schematic representation showing the organization of FPV and amino acid mutations in the VP2 protein. The FPV strains explored herein are colored with red. Capital letters represent amino acids.

Moreover, an analysis was conducted to compare the amino acid sequences of VP2. The amino acid sites 80/93/103/323/568 in all 36 sample sequences were found to be entirely in agreement with the characteristic amino acid sites of FPV, as depicted in Figure 3 (28). In comparison to FPV prototype CU-4 (GenBank accession number M38246), mutations were mainly concentrated in 6 positions (A91S, I101T, I145L, V232I, A300P, and V562L), where the most common mutations were A91S (67.6%) and I101T (73.0%).

### 3.3 Genetic evolution and amino acid mutations of NS1

Since non-structural proteins are important in virus replication and pathogenicity, their phylogeny and amino acid mutations were investigated as well. Phylogenetically, 36 NS1 nucleotide sequences from this study and 38 reference sequences from GenBank formed three different groups, G1, G2 and G3 (Supplementary Figure S1). The G1 group mainly comprised strains from China and Japan, and contained 18 (50%) of the isolated strains in this study. The G2 group consisted exclusively of Chinese isolates, with 18 (50%) of the isolated strains in this study. G1 and G2 branches originated from a single node, suggesting the NS1 sequences of the strains isolated in this study is limited (Supplementary Figure S1).

Compared to FPV prototype CU-4, amino acid sequence analysis demonstrated that the mutation sites were concentrated in 12 sites (N23D, E72K, E73K, S136R, R148H, K201R, H247Q, T248I, I574V, H595Q, V596L and D616N). Among these, the most common mutations detected were H247Q (45.9%), T248I (48.6%), H595Q (91.9%) and V596L (91.9%) (Supplementary Figure S2). The biological significance of these mutation sites needs to be investigated further.

### 3.4 Viral titers and growth characteristics of FPV strains

**Figure 4:**
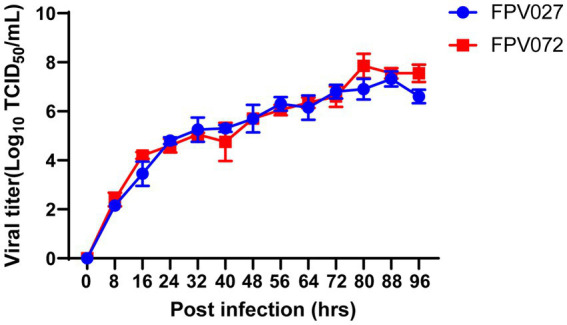
Single growth curve analysis of CRFK cells infected with FPV027 and FPV072 strains over 96 h.

To analyze the replication characteristics of FPV strains, the virus titers were calculated in a defined time. CRFK cells were infected with the FPV strain at an MOI of 0.1. Then, the supernatants were harvested at 80 h after induction, and the virus titers in the samples were determined. As shown in Table 2, the viral titers ranged from 2.7 ± 0.6 log_10_ TCID_50_/mL to 7.2 ± 0.3 log_10_ TCID_50_/mL. Among them, the titers of FPV072 were highest with 7.2 ± 0.3 log_10_ TCID_50_/mL, followed by FPV027, with 6.9 ± 0.5 log_10_ TCID_50_/mL. In addition, the growth characteristics of FPV072 and FPV027 were studied using one-step growth experiments. CRFK cells were inoculated with FPV strains and the supernatants were collected every 8 h post-infection to determine viral titers. As shown in Figure 4, viral growth of FPV027 and FPV072 reached peak titer 80–88 h post-infection and declined thereafter. No significant difference was found between the two strains.

**Table 2:** The virus titer of isolated strains.

FPV Strain	Virus titer (log_10_ TCID_50_/mL)^a^	Group	FPV strain	Virus titer (log_10_ TCID_50_/mL)	Group
FPV072	7.2 ± 0.3	G1	FPV049	6.2 ± 0.4	G2
FPV027	6.9 ± 0.5	G2	FPV037	6.2 ± 0.3	G1
FPV013	6.8 ± 0.6	G2	FPV041	6.1 ± 0.6	G1
FPV055	6.8 ± 0.5	G1	FPV089	6.1 ± 0.4	G1
FPV044	6.8 ± 0.3	G1	FPV003	6 ± 0.3	G2
FPV088	6.8 ± 0.5	G1	FPV043	5.9 ± 0.5	G1
FPV086	6.7 ± 0.6	G1	FPV029	5.8 ± 0.6	G1
FPV026	6.6 ± 0.4	G2	FPV090	5.8 ± 0.4	G1
FPV046	6.5 ± 0.5	G2	FPV080	5.8 ± 0.4	G1
FPV028	6.5 ± 0.3	G2	FPV038	5.74 ± 0.4	G1
FPV087	6.5 ± 0.4	G1	FPV030	5.7 ± 0.6	G1
FPV048	6.4 ± 0.6	G2	FPV076	5.7 ± 0.3	G1
FPV036	6.4 ± 0.5	G1	FPV085	5.1 ± 0.5	G1
FPV045	6.4 ± 0.3	G1	FPV077	5.1 ± 0.4	G1
FPV021	6.3 ± 0.6	G2	FPV014	4.7 ± 0.6	G2
FPV042	6.3 ± 0.5	G1	FPV050	4.7 ± 0.6	G2
FPV081	6.3 ± 0.5	G1	FPV040	3.75 ± 0.8	G1
FPV008	6.2 ± 0.4	G2	FPV039	2.7 ± 0.6	G1

a The data were obtained from three independent parallel experiments, and the data were presented as mean ± SD.

### 3.5 Pathogenicity of the FPV 027 and FPV072 strains

**Figure 5:**
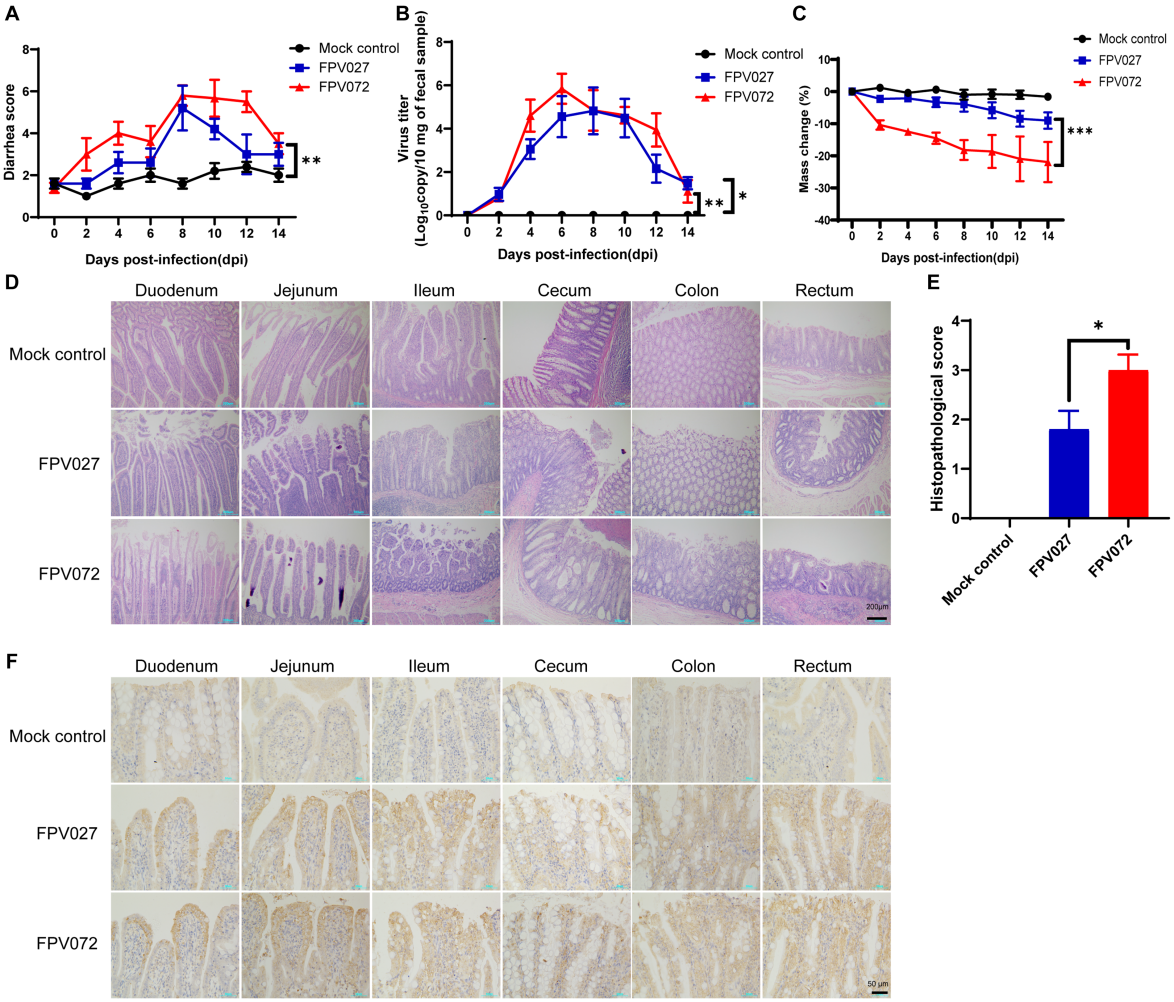
Diarrhea score, virus shedding in feces, weight loss, histopathology and immunohistochemistry (IHC) of cats inoculated with FPV. **(A)** Diarrhea score in cats after FPV inoculation. **(B)** Virus shedding in feces. **(C)** Weight loss of cats. **(D,E)** Intestinal histopathology and score in FPV-injected and negative control cats. **(F)** Detection of FPV antigen by IHC analysis of intestinal tissue sections from FPV infected and mock control (400× magnification). Data was processed based on the results of each group (*n* = 5).

As previously mentioned, the phylogenetic tree, which was constructed using VP2 sequences of these 36 FPV strains, was categorized into two groups, namely G1 and G2. However, it remains unknown if there are differences in pathogenicity between viruses in the two groups. Then, two representative strains with high titers and from different evolutionary branches and regions (FPV072: 7.2 ± 0.3 log_10_ TCID_50_/mL, Hunan; FPV027: 6.9 ± 0.5 log_10_ TCID_50_/mL, Shanghai) were chosen to conduct further studies on pathogenicity comparison. Cats were challenged with 10^4.7^ TCID_50_ FPV strains and the clinical signs were monitored and compared daily. As shown in Table 3, the number of total WBCs significantly declined 4–6 days post-inoculation, and death occurred when it dropped to below 2 × 10^9^/L. In addition, WBCs were significantly lower in FPV072 infected cats than in FPV027 infected cats. According to the fecal scoring system (Supplementary Table S2), cats infected with the FPV072 strain developed higher fecal scores on days 2, 4, 10 and 12, indicating more severe leukopenia diarrhea in FPV072-infected cats (Figure 5A). There was no significant difference in FPV shedding in feces between two groups (Figure 5B). Compared with those infected with FPV027, the weight loss of mice infected with FPV072 was more obvious (Figure 5C).

**Table 3:** Changes in WBC counts in the FPV infection groups and mock control group.

FPV strains	Cat number	WBC Count (10^9^/L)			
		0 dpi	2 dpi	4 dpi	6 dpi
FPV027	1	15.20	11.95	10.96	6.29
	2	8.52	8.76	6.48	6.34
	3	13.62	13.74	9.28	7.48
	4	19.22	22.97	19.76	20.01
	5	9.76	10.88	9.36	7.90
FPV072	1	7.58	7.50	5.34	1.64
	2	11.66	11.49	6.28	6.04
	3	10.58	9.46	8.72	7.60
	4	14.76	12.01	10.76	6.58
	5	8.90	8.72	6.60	6.50
Mock control	1	15.02	14.64	14.73	15.37
	2	27.84	28.42	29.43	28.93
	3	13.44	13.48	13.25	13.08
	4	8.50	8.39	8.48	8.53
	5	12.48	12.50	12.37	12.18

Histopathological examination showed prominent changes, including villous necrosis of the intestine, mucosal erosion and ulceration of the cecum colon and rectum. A higher intestinal histopathological score in the FPV072 group indicated more severe intestinal damage (Figures 5D,E). Furthermore, immunohistochemical examinations revealed that FPV antigens were predominant in the cytoplasm of the epithelial cells of atrophied villi in the small intestines (Figure 5F). Thus, cats in the FPV072 group showed more severe pathological changes. In addition, enlarged and mottled spleens were also found in a minority of infected cats (Supplementary Figure S3A). On 14 dpi, spleens of infected mice exhibited significant congestion and an increase in size of the red and white pulp (Supplementary Figure S3B).

### 3.6 Comparison of VP2 and NS1 protein structures between FPV027 and FPV072 strains

**Figure 6:**
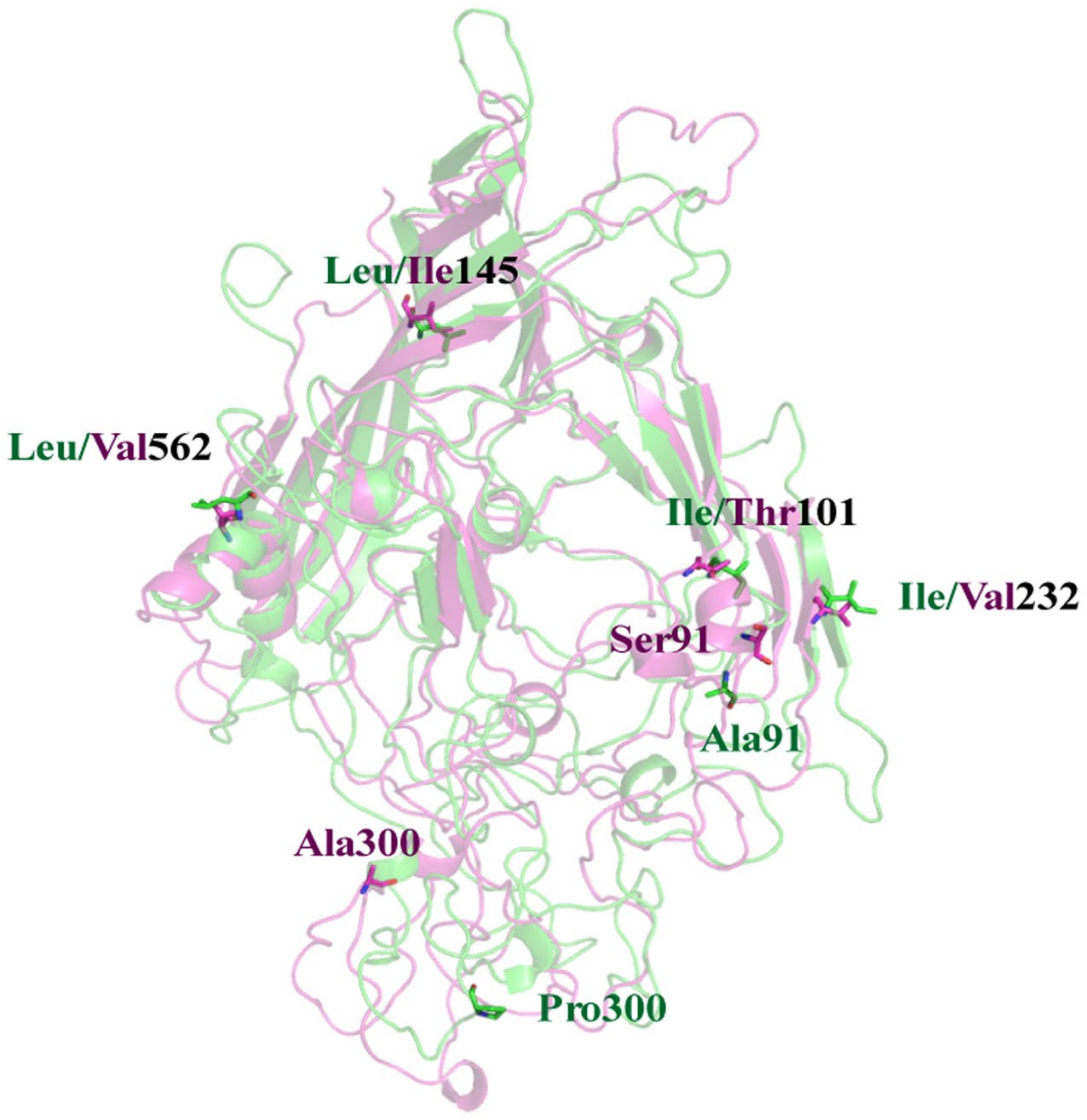
A superposition of FPV027 VP2 (green) and FPV072 VP2 (magentas). Mutated residues are shown in sticks. The residue numbers are labeled nearby with the corresponding color.

As previously mentioned, there exists a disparity in the pathogenic potential between the FPV072 and FPV027 virus strains, which cannot be attributed to host-specific variations. Hence, we tried to elucidate this distinction from the perspective of viral molecular protein structures. A 200 ns MD simulation was performed for the VP2 protein of each virus. The representative conformations obtained from the cluster analysis of the two systems are superimposed. As shown in Figure 6, the mutation of residues at 101, 145, and 562 site have no significant effect on the structure. It’s worth noting that Ser91 is part of the alpha helix in FPV072 VP2, whereas Ala91 has no stable secondary structure in FPV027 VP2. This change also induces a certain bend in the β-sheet where residue 232 located. Besides, due to the mutation of residue 300, a large conformational shift occurs near it. The above conformation changes may affect the virulence of the virus.

**Figure 7:**
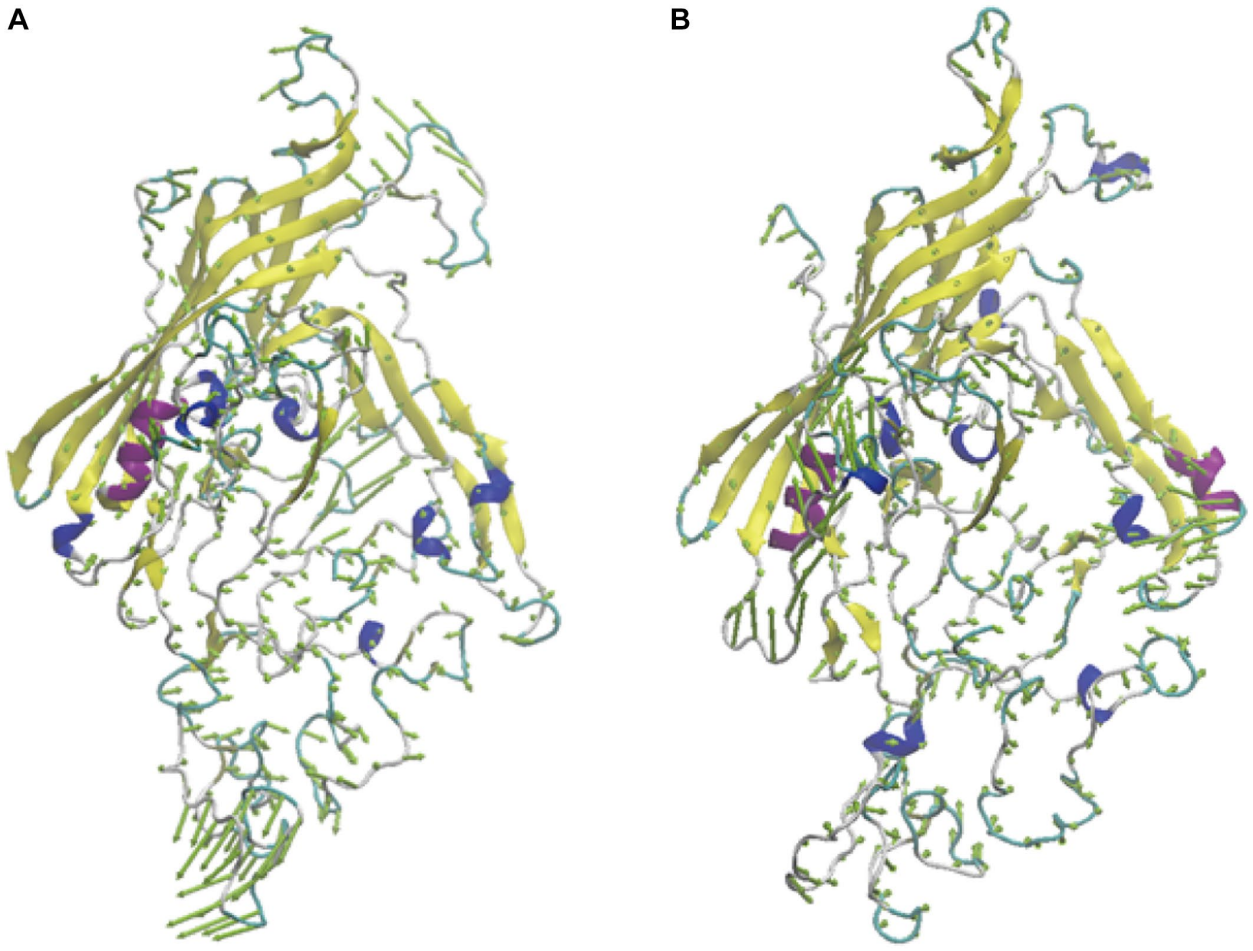
The porcupine plots of system FPV027VP2 **(A)** and FPV072 VP2 **(B)**.

To further analyze the effect of mutations on protein movement patterns, we performed principal component analysis of these two proteins and presented the results in the form of porcupine diagrams. Our main emphasis in this section will be on the principal modes derived from the highest eigenvalue (PC1) and its corresponding eigenvector. In Figure 7, the spine direction represents the eigenvector, and the length of the spine represents the eigenvalue. There are different motion modes in the two different systems, which could be the reason for the difference in infectivity and pathogenicity.

## 4 Discussion

The feline parvovirus infection is a prevalent ailment that induces diarrhea in cats and can be life-threatening in severe cases. In recent years, the prevalence of FPV infection remains high in different countries and regions. A findings of a study conducted in central and eastern China from 2018 to 2022 revealed that 27.6% of cats with clinical gastroenteritis were infected with FPV (29). While, the prevalence of FPV in southwest China from 2019 to 2021 was 56.7% (30). Here, a total of 167 FPV positive specimens were obtained during a single quarterly period from 8 veterinary hospitals in different aeras, indicating the FPV positive cats in China was considerable. Furthermore, the prevalence of FPV exhibits regional variations. It was reported that the prevalence positivity of FPV in Italy, Egypt, and Spain are 73.5%, 43%, and 18%, respectively (31–33). The investigation into the epidemiological characteristics, pathogenic features, and underlying mechanisms of FPV will establish a fundamental framework for the prevention and control strategies against FPV.

In this work, 36 FPV strains were isolated from different parts of China in 2021, and their molecular prevalence was investigated. The phylogenetic tree based on VP2 or NS1 genes showed that all the isolated strains were distributed on two branches. Two genetically different FPV strains (027 and 072) from each branch were chosen to compare pathogenicity in a domestic cat infection model, showing differences in degree of infectivity and pathogenicity. While individual variations in feline characteristics may account for these disparities, the pathogenicity is also influenced by mutations occurring in viral proteins. Comparison of sequences in the two strains showed 6 amino acid substitutions in VP2, at positions 91, 101, 145, 232, 300 and 562 (Figure 6). Residue 91 is located within the LOOP1 region, contradicting the assertion that the LOOP1 segment is highly conserved (34). The residue is situated close to residue 93, positioned at the apex of the capsid’s 3-fold spike. It is among the residues that play a role in determining the range of hosts, although the precise phenotypic impact of these mutations remains uncertain. It has been suggested that mutation at residue 91 increases strain virulence, in agreement with our pathogenicity test results (35). Additional research has suggested that residue 101 is situated not only within the central region of the receptor-binding area (36) but also within the site where antibody bind (37). Structural data has also shown that a Thr at position 101 leads to the formation of a polar contact with Asp99. The modification alters the outer layer of the central area responsible for binding to receptors, thereby affecting the strength of the bond between the transferrin receptor and the virus (11). Moreover, a mutation at position 101 may cause immune failure (38), which may also explain why the current vaccine is only partially effective. Since residues 299–301 on LOOP3 are part of neutralizing site B, a mutation at residue 300 may affect antigenicity (39).

The NS1 protein has crucial functions in regulating replication, DNA packaging, cytotoxicity, and pathogenicity. Three substitutions in the NS1 coding region, at positions 247, 248 and 574, were also found (data not shown). Mutations at positions 247 and 248 were also found in mink enteritis virus (40). Mutation at position 574 of the NS1 protein has not been reported previously in FPV. Additional research is necessary to ascertain its importance in the contagion of cats.

Our phylogenetic tree and homology analysis revealed that all the 36 FPV strains isolated clustered into two branches, where genogroup G1 contains most (24/36, 66.7%) of the isolates and G2 contains a smaller fraction (12/36, 33.3%). Notably, two vaccine strains were clustered within the G2 group, and they were distantly related to the currently prevalent strains, which may explain the low protective effect of the current vaccine. The development of vaccines utilizing newly circulating strains is imperative.

The incubation period of FPV is generally less than 14 days (5), and the median time from shelter admission to FPL diagnosis is 9 days (41). The risk of acquiring FPV infection is even higher in the stressful environment of shelters (42). In our study, domestic cats were maintained under well-controlled and standardized barrier conditions for 14 days followed by antigen and serology tests to screen cats that had been previously infected with FPV or in the incubation period. Nonetheless, after the observation period, a minority of non-inoculated cats showed clinical symptoms of FPV infection, indicating possible infection by direct fecal-oral contact during the observation period. Since the experimental cats used in this study were 8–12 weeks of age, the balance period could not be extended and this may add some uncertainty to our data.

It has been reported that FPV mainly replicates in small intestinal crypt cells and lymphoid cells (43). Replication often leads to bleeding in the small intestine and stomach, which is the main cause of hemorrhagic diarrhea in cats (41). However, in the present study, no hemorrhagic diarrhea was observed. This discrepancy may be caused by different doses and strains of FPV. Other lesions were also detected, such as enlarged spleen and lymph nodes in the mesentery. Despite splenomegaly is rarely described in clinical symptoms of FPV infection, about one-third of cats exhibited splenomegaly with rough spleen surfaces, possibly due to antiviral host responses, such as antibody synthesis and lymphocyte proliferation (44).

## 5 Conclusion

Based on phylogenetic analysis, the 36 strains isolated in China in 2021 were classified into two groups, G1 and G2, with most strains clustering in the G1. G1 group isolate FPV072 exhibits higher infectivity and pathogenicity than G2 group isolate FPV027. Structural alignment of the VP2 protein of the two viruses showed that there were mutations of residues at 91, 232, and 300, which might be related to the difference in infectivity and pathogenicity between the two groups. These observations will be helpful for better understanding the prevalence of FPV infection in China and the development of an FPV vaccine.

## Data availability statement

The datasets presented in this study can be found in online repositories. The names of the repository/repositories and accession number(s) can be found in the article/Supplementary material.

## Ethics statement

The animal study was approved by Institutional Animal Care and Use Committee (IACUC) of Ludong University. The study was conducted in accordance with the local legislation and institutional requirements.

## Author contributions

QX: Methodology, Writing – original draft. ZS: Methodology. XX: Validation. YP: Methodology. SZ: Methodology. YL: Investigation. JiuZ: Formal Analysis. LJ: Visualization. JiaZ: Resources. HZ: Resources. XY: Project administration, Writing – review & editing. XZ: Funding acquisition.

## Footnotes

^1^ https://yanglab.qd.sdu.edu.cn/trRosetta/
