# Supplementary material for: China-origin G1 group isolate FPV072 exhibits higher infectivity and pathogenicity than G2 group isolate FPV027
Source: Front Vet Sci. 2024 Jan 15;11:1328244. doi: 10.3389/fvets.2024.1328244 (PMC10822907; doi:10.3389/fvets.2024.1328244)
Supplement: Supplementary file 1 [file Data_Sheet_1.docx]

Supplementary Materials

**China-origin** **G1 group isolate FPV072 exhibits higher infectivity and pathogenicity than G2 group isolate FPV027**

**Qiaoqiao Xie^1, 2, 3^, Zhen Sun^1, 2, 3^, Xiu Xue^1, 2, 3^, Yajie Pan^1, 2, 3^, Shuye Zhen^1, 2, 3^, Yang Liu^1, 2, 3^, Jiuyu Zhan^1^, Linlin Jiang^1, 2, 3^, Jianlong Zhang^1, 2, 3^**

*** Correspondence:**

Hongwei Zhu^1, 2, 3, ⁎^, Xin Yu^1, 2, 3,^ ^⁎^, Xingxiao Zhang^1, 2, 3,^ ^⁎^

E-mail addresses: hngwzhu@outlook.com (H. Zhu), yuxinzghn@ldu.edu.cn (X. Yu), zhangxingxiao@ldu.edu.cn (X. Zhang).


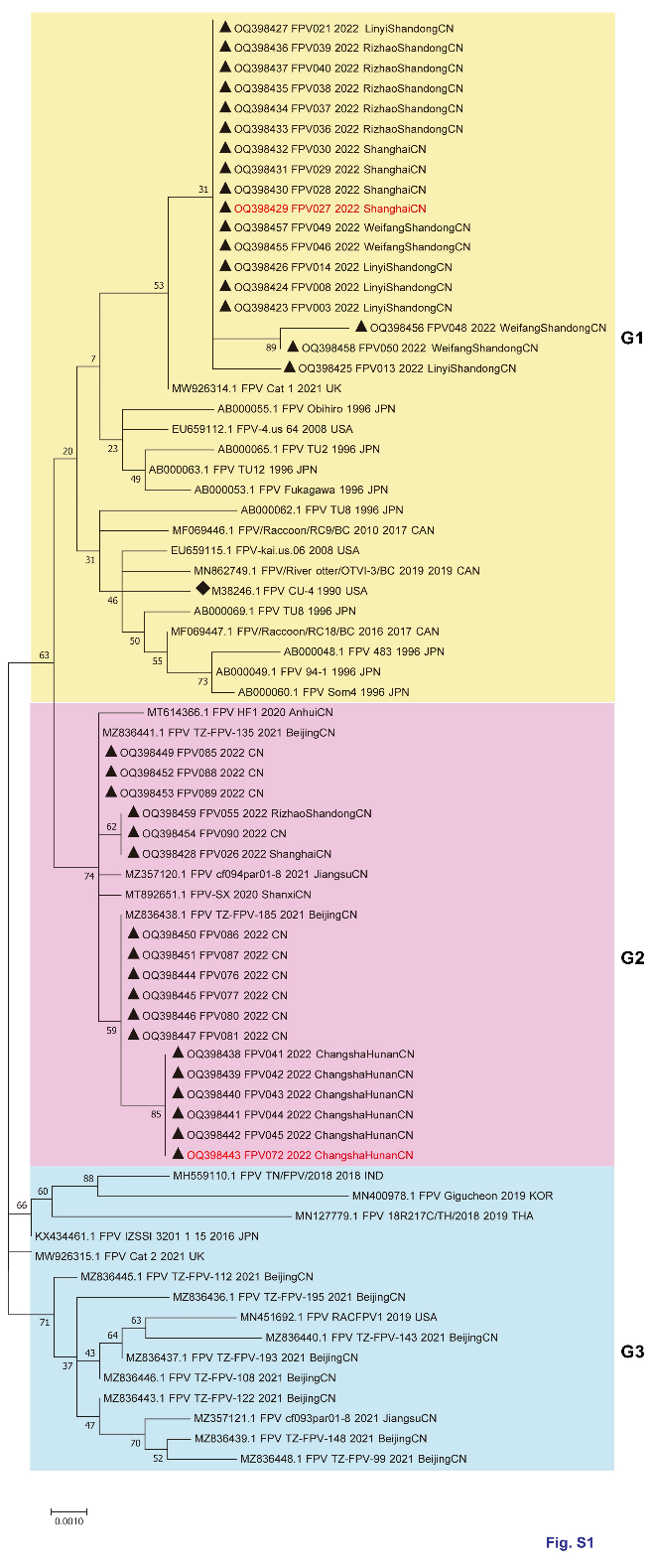
**Supplementary Figures**

**Supplementary Figure S1**. Phylogenetic comparison of NS1 nucleotide sequences in FPV isolates and those found in the GenBank database. Horizontal branch lengths are proportional to genetic distances. Scale bars indicate nucleotide substitutions per site. Red text indicates FPV strains explored herein, ◆indicates FPV standard strain, ▲indicates FPV isolate strain; FPV cluster 1(G1), 2(G2) and 3(G3) are shown with different background colors.


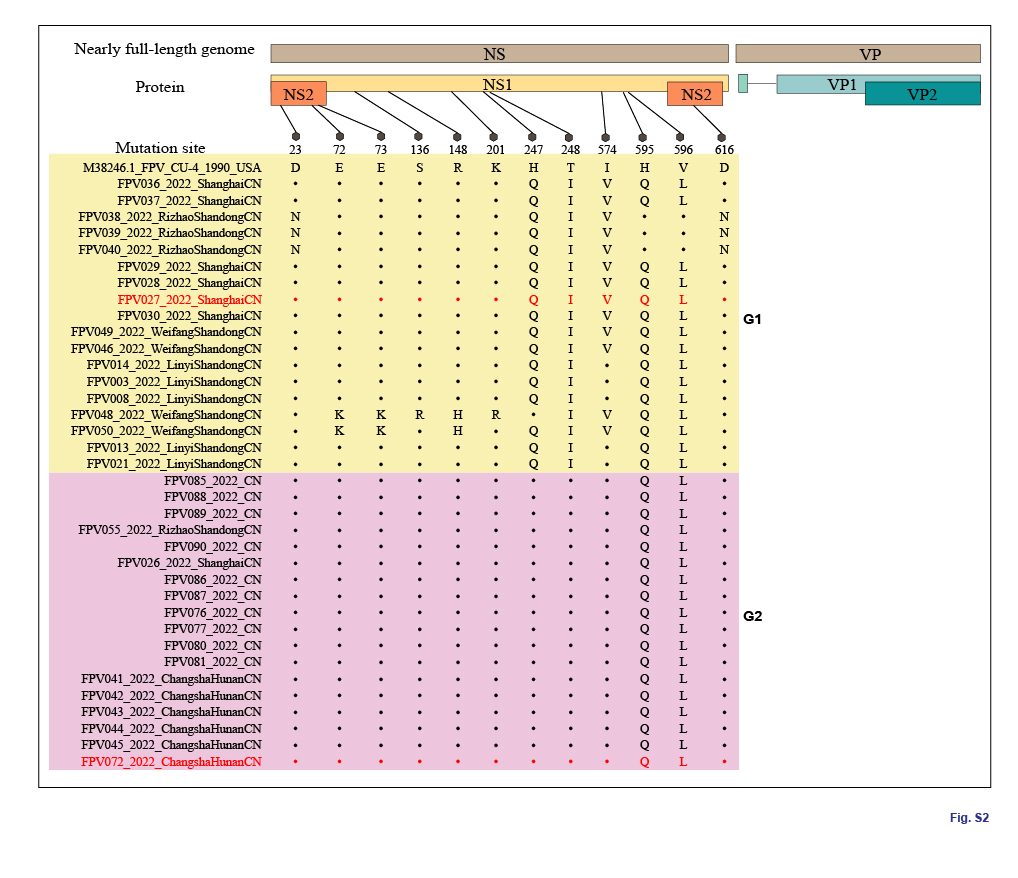


**Supplementary Figure S2.** Molecular schematic representation showing the organization of FPV, amino acid mutations in the NS1 protein of FPV. Capital letters represent amino acids.


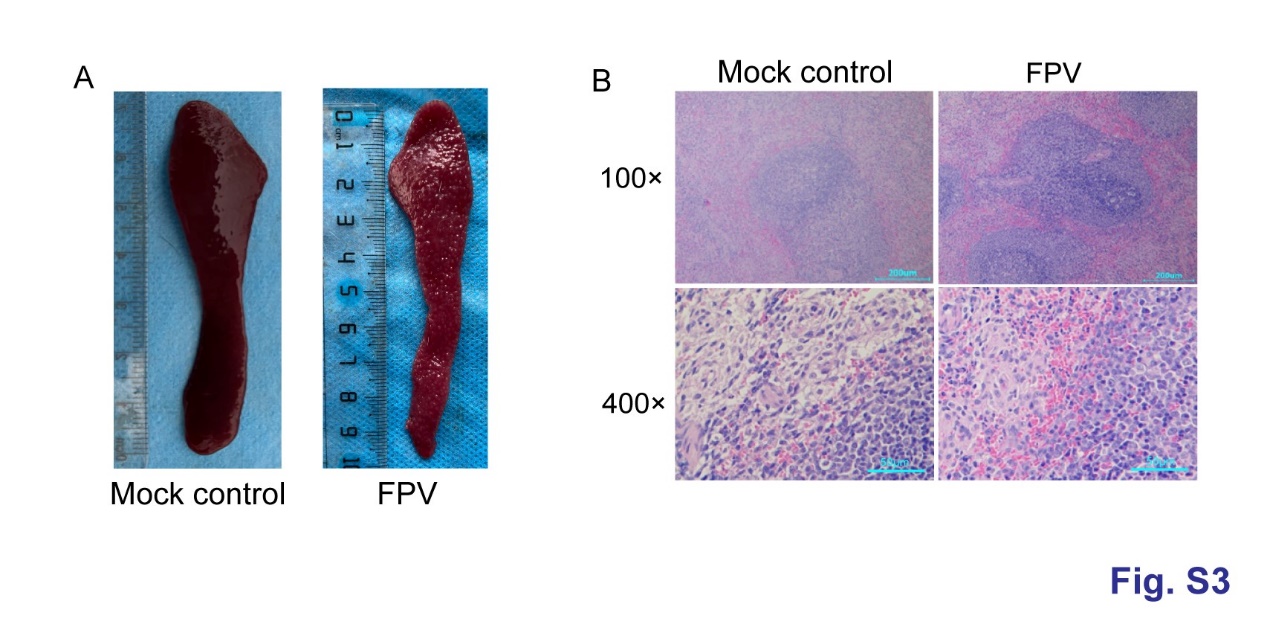
**Supplementary Figure S3.** Spleen morphology and histopathology of cats inoculated with FPV. (A) Spleen morphology of negative control cats and FPV challenged cats on 14 dpi; (B) Spleen histopathology of negative control cats and FPV challenged cats on 14 dpi.

**Supplementary Tables**

**Supplementary Table S1.** PCR primers used for amplification of the VP2 and NS1 genes of FPV

| Primer | Sequence (5’ to 3’) | ^a^ Location(bp) | ^a^ Size (bp) |
| --- | --- | --- | --- |
| FPV-F | AAAGAGTAGTTGTAAATAA | 3024-3043 | 681 |
| FPV-R | TATATCACCAAAGTTAGTAG | 3685-3704 |  |
| VP2-F | GGTCCGAAATAGAGGCAGAC | 2200-2219 | 2396 |
| VP2-R | GGTGCTAGTTGATATGTAATAAACA | 4561-4585 |  |
| NS1-F | CGCTTCTTGTCTTTGACAGAGTGAACCTCTCTTAC | 227-261 | 2100 |
| NS1-R | TCTTTGCCGGAGGTGCCATCGTACC | 2280-2304 |  |

^a^ The size and nucleotide position of the PCR products, according to the genomic sequence of FPV (GenBank accession No. M38246)

**Supplementary Table S2.** Fecal scoring system.

| Description of fecal morphology | Score | Fecal morphology |
| --- | --- | --- |
| Very hard and dry; requires much effort to expel from the body; no residue left on the ground when picked up. Often expelled as individual pellets. | 1 | 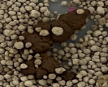 |
| Firm, but not hard; should be pliable; segmented appearance; little or no residue left on the ground when picked up. | 2 | 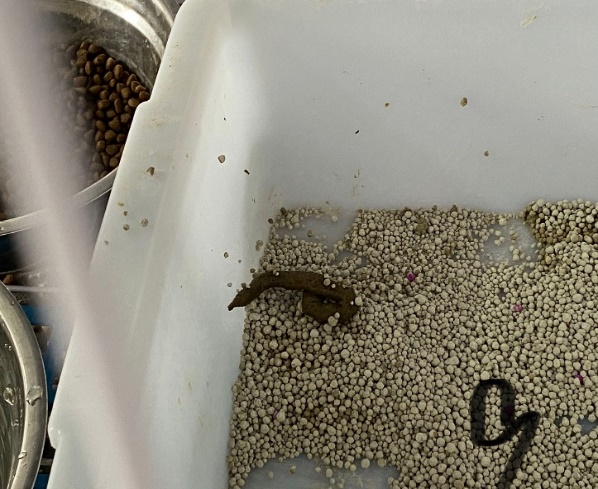 |
| Log-like; little or no segmentation visible; moist surface; leaves residue, but holds form when picked up. | 3 | 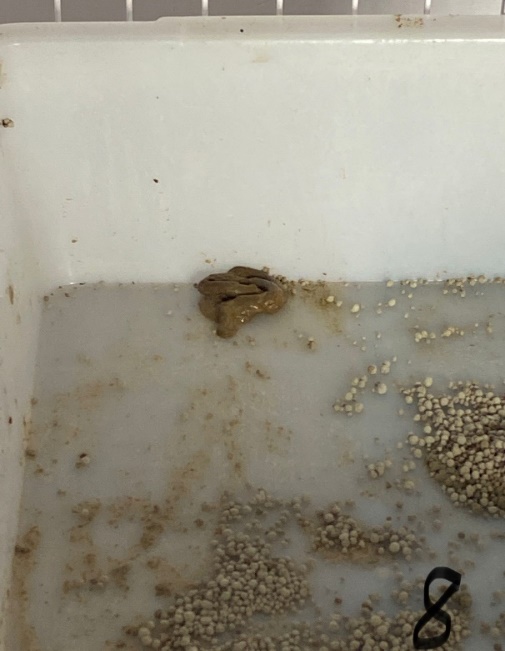 |
| Very moist (soggy); distinct log shape visible; leaves residue and loses form when picked up. | 4 | 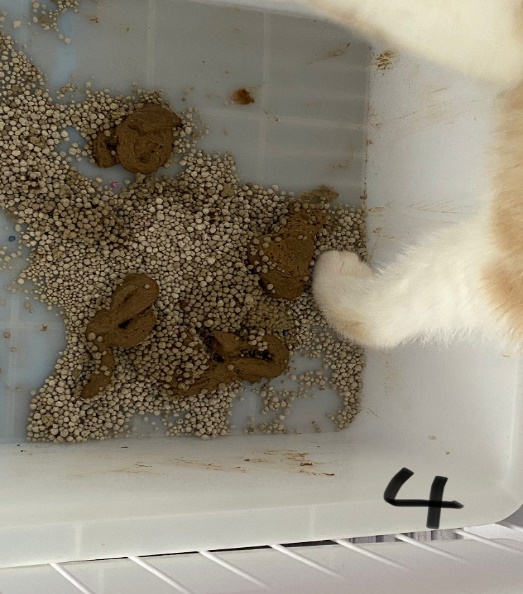 |
| Very moist but has a distinct shape; present in piles rather than as distinct logs; leaves residue and loses form when picked up. | 5 | 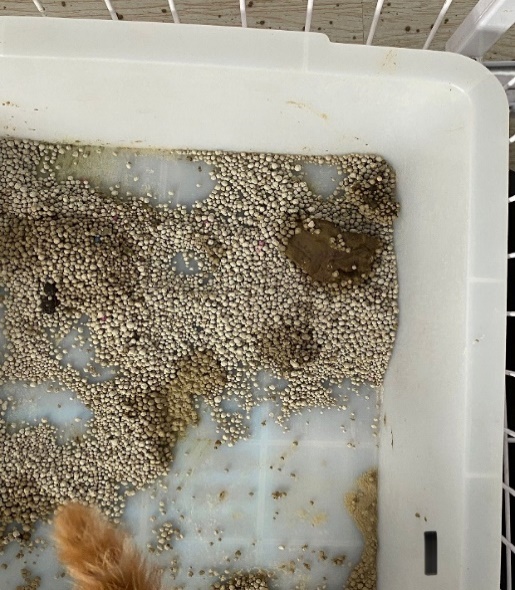 |
| Has texture, but no defined shape; occurs as piles or as spots; leaves residue when picked up. | 6 | 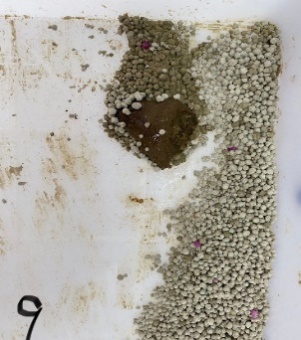 |
| Watery, no texture, flat; occurs as puddles. | 7 | 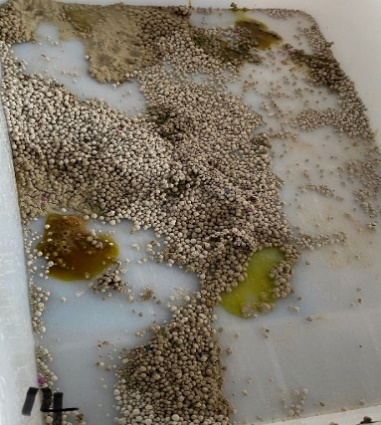 |
